# Department of Practice of Medicine

**Published:** 1893-08

**Authors:** Allen J. Smith

**Affiliations:** Medical Department University of Texas, Galveston


### Current Medical Literature.

### DEPARTMENT OF PRACTICE OF MEDICINE.

Conducted by Professor Allen J. Smith, A. M., M. D., Medical Department University of Texas, Galveston.

DIPHTHERIA TREATED BY THE TARTARIC ACID CORROSIVE SUBLIMATE OF MERCURY METHOD.—M. G. Tull, of Philadelphia (*Times and Register*, May 20, 1893), attracted by the report of

Rennert that he had found a 1-500 tartaric acid corrosive sublimate solution, swabbed on the faucial walls every six hours, the most valuable local application to the diphtheritic membrane, made use of the same plan in a series of cases, with excellent results. In a group of between forty and fifty cases Dr. Tull met in the autumn of 1889, he lost no case. During the autumn and winter of 1892, a second series of thirty-three cases was encountered, in the Baptist Orphanage of Philadelphia. The first nine cases were at once isolated, and the following treatment employed in a routine manner in each case. Each child was given one of the following triturates every half hour until a free movement of the bowels had been secured, and after that every two hours:

℞ Calomel ........................ gr. 1-5
Sodium bicarbonate ................ gr. j
Ipecacuanha ................... gr. 1-10

The following mixture, modified to suit the ages of the children, was also employed:

℞ Quinine sulphate.
Potassium chlorate.
Potassium citrate.
Syrup of the chloride of iron.
Syrup of yerba santa.
M. Sig.: A dose every two hours.

Nourishment consisted of liquid peptones (S. & D.) at the commencement, rapidly followed by a full diet as the patient convalesced. Every three hours the throat was swabbed, an ordinary pledget of cotton being used in the application, with the 1-500 solution of tartaric acid corrosive sublimate (Mulford's tablet, containing 3-85 gr. of bichloride of mercury and 19.25 gr. of tartaric acid in a gill of water). The results were immediate, and the patients all recovered rapidly, so rapidly, in fact, that it was believed by the house attendants that the sickness was not diphtheria, and in consequence of this disbelief, one self-sufficient matron purposely neglected calling attention to five new cases, which she proposed treating herself, to prove that the physician was wrong. She concealed the cases until the disease had progressed some days, and until she became alarmed at their severity. Of these five, three died, and following these a second outbreak occurred, eighteen fresh cases, who had been imperfectly isolated from the five, appearing. Of the last number, two died, one a child of four years of age, of exceedingly bad family history, and evidently a weakling, the second from a severe intercurrent attack of measles. All the rest were promptly benefitted by the course of treatment pursued. Dr. Tull urges the propriety of the method, in that from experimentation Laplace had a number of years since proved that there is a strong antagonism exerted by this acid sublimate solution upon the diphtheritic bacillus.

---

PATHOLOGY AND TREATMENT OF GRAVES' DISEASE (EXOPHTHALMIC GOITRE).—Dr. W. H. Thompson, of New York (*N. Y. Med. Journal*, June 3, 1893), calls especial attention to the frequent absence of either or both the exophthalmos and the goitre in Graves' disease, and remarks the inaccuracy of its common name. He believes the tachycardia and the tremor are the two most constant symptoms, and suggests as the point of origin of the various manifestations of the affection, the nuclear origin of the glosso-pharyngeal, the vagus and spinal accessory nerves, a paralytic lesion being capable of accounting for the different symptoms. The author acknowledges there can be little structural alteration in this region, without involvement of the adjacent vital spots, but relies upon the supposition that through digestive failures toxic substances are formed, and are carried about through the circulation and exhibit a selective power upon these parts of the nervous system. In conformity with this idea, Dr. Thompson takes especial care to look after the intestinal digestion in all cases of exophthalmic goitre, cutting down the meat supply and substituting fermented milk. Upon such diet, and with very little medication, the author claims to have met exceedingly good results from his treatment.

---

TUBERCULOSIS IN THE NEGRO.—J. A. Pritchett (*Alabama Medical and Surgical Age*, June, 1893), in a paper recently read before the Alabama State Medical Association, discusses with interest the question of the generally admitted increase of tuberculosis in the negro since the war. Dr. Pritchett ascribes—as in fact do almost all physicians whose experience has extended over a sufficient period of years in the South—the greatest influence in this increase to the altered relations of life encountered by the black after his emancipation. The careful isolation of the negro upon the plantation; the denial of free intercourse with the neighboring plantations or towns; the forced regularity of his hours and meals; the plain, nourishing quality of his food; the care exercised over the sick; the vigilance in the marriage regulations, looking to the physical improvement of the offspring; the care as to the cleanliness and drainage in the quarters; the denial of excessive excitement, religious, political, or otherwise,—all these contributed to the well-being of the slave upon the aveage plantation in ante bellum days. In the present days, nearly all these conditions are reversed for the majority of the race, and of necessity the opportunities for infection by tuberculosis have increased manifold in consequence of the change. The improper clothing, the improper food, the greatly increased possibilities of exposure of every sort, have largely contributed to the acquirement of those catarrhal conditions affording the predisposition most favorable to tubercular infection. So, too, all these factors influence the acquirement of other infectious diseases, to which the slave was apparently almost immuned—the fevers, and syphilis. The writer lays especial stress to the great frequency of phthisis florida in the negro, the rapid and almost unheralded course of the disease, and the great likelihood of mistaking this for an ordinary uncomplicated croupous pneumonia until the failure of the usual periods in the pneumonia suggests the possibility of the presence of the fatal infection.

---

REST VS. EXERCISE IN THE TREATMENT OF PULMONARY CONSUMPTION.—Dr. T. J. Mays (*Medical and Surgical Reporter*, June 17, 1893), calls attention, in a recent most valuable article, to the great worth of rest, absolute or partial, in the treatment of phthisis. The old and very prevalent idea that strength is to be gained by consumptives by exercise in the open air, is perhaps not to be questioned as the patient approaches near to the strength of a healthy individual; but the weak and shattered individual, with resistive power already much below normal, is not strengthened, but on the contrary, is further weakened by the extra exertions thrown upon him. The author impresses his teaching by the simile of a financier, who, with full treasury, may attempt the greatest financial feats with confidence in the results, but who with lowered exchequer attempts even less daring speculations only with the greatest risk of absolute monetary dissolution. Dr. Mays would prescribe, as one of the most essential features in the treatment of the incipient cases, where loss of strength and of flesh is perhaps the only marked symptom, and in the very weak and emaciated case, a course of rest in bed,—or if not rest in bed absolutely, at least pronounced bodily rest, with relief from all the ordinary cares of life, and all daily exercises. With this as a prime point in his treatment, combining it with the usual care in nutrition, the author has demonstrated, upon a series of cases which he quotes in the article in question, a marked improvement with increase in weight and strength in almost every case, in some instances entire relief from symptoms.

